# Factors influencing outcomes after medial hamstring lengthening with semitendinosus transfer in patients with cerebral palsy

**DOI:** 10.1186/s12984-017-0296-0

**Published:** 2017-08-14

**Authors:** Ki Hyuk Sung, Jaebong Lee, Chin Youb Chung, Kyoung Min Lee, Byung Chae Cho, Seung Jun Moon, Jaeyoung Kim, Moon Seok Park

**Affiliations:** 1 Department of Orthopaedic Surgery, Seoul National University Bundang Hospital, 82 Gumi-ro 173 Beon-gil, Bundang-Gu, Seongnam, Gyeonggi South Korea; 2 Medical Research Collaborating Center, Seoul National University Bundang Hospital, 82 Gumi-ro 173 Beon-gil, Bundang-Gu, Seongnam, Gyeonggi South Korea; 3 Department of Orthopaedic Surgery, Seoul Jaeil Hospital, 70 Jisan-ro, Pyeongtack, Gyeonggi South Korea; 4 Department of Orthopaedic Surgery, H Plus Yangji Hospital, 1636 Nambusunhwan-ro, Gwanak-Gu, Seoul, South Korea

**Keywords:** Distal hamstring lengthening, Cerebral palsy, Flexed knee gait

## Abstract

**Background:**

Although several studies have investigated the outcomes after distal hamstring lengthening (DHL), no study has undertaken an approach that included all or most of the important factors that could influence the results. This study was performed to evaluate the outcomes after DHL and analyze the factors that influence the improvement and serial change in knee motion after surgery in patients with cerebral palsy (CP), using a linear mixed model (LMM).

**Methods:**

The study included 314 ambulatory CP patients (594 limbs) with spsastic diplegia who were followed up after undergoing DHL as part of a single-event multilevel surgery and who underwent preoperative and postoperative 3-dimensional (3D) gait analyses. Relevant kinematic values, including knee flexion at initial contact, minimum knee flexion in the stance phase, knee range of motion (ROM), mean pelvic tilt and gait deviation index (GDI) score, were the outcome measures. Changes in knee motion and the GDI score were adjusted for multiple factors, such as sex, the Gross Motor Function Classification System (GMFCS) level, and concomitant surgeries as fixed effects, and follow-up duration, laterality, and each subject as random effects, using a LMM.

**Results:**

We found significant improvements in knee flexion at initial contact, minimum knee flexion in the stance phase, knee ROM, and GDI score 2 years after DHL. In patients with GMFCS level I and II, improvement in all sagittal knee kinematics was maintained during follow-up. In addition, GDI score, which represents overall gait pathology, consistently improved throughout the follow-up duration (1.2 per year, *p* = 0.008).

**Conclusion:**

Medial hamstring lengthening with semitendinosus transfer, as a part of a SEMLS, was effective procedure in treating flexed knee gait with regard to sagittal knee kinematics and GDI score in spastic CP with flexed knee gait.

## Background

Flexed knee gait is one of the most common gait abnormalities in patients with cerebral palsy (CP), and it is partly caused by spastic and contracted hamstring muscles [1]. Distal hamstring lengthening (DHL), as part of a single-event multilevel surgery (SEMLS), is widely considered the standard surgical procedure for the correction of increased knee flexion in patients with CP [2, 3].

Previous studies have shown that DHL is effective for reducing knee flexion and improving knee motion [2, 4–16]. However, there have been concerns that DHL might aggravate anterior pelvic tilt, lumbar hyperlordosis, and genu recurvatum, and eventually induce crouch gait [2, 17]. Even though the length of time of improvement in knee motion are maintained is unclear, several studies showed that the improvements after DHL were maintained 10 years postoperatively in patients with CP [11, 13, 14, 18].

Limb-based analysis could cause demographic data to be duplicated [8]. Additionally, the inclusion of both limbs in statistical analyses violates the underlying principle of statistical independence and could bias the study results by exaggerating the significance levels and narrowing the confidence intervals if the findings for the limbs of an individual are significantly related to each other [8, 19, 20]. However, most previous studies including bilateral cases did not consider this issue. Moreover, previous studies have not considered factors that could affect the study results, such as age, sex, the anatomical type of CP, Gross Motor Function Classification System (GMFCS) levels, and concomitant surgeries. These factors should be considered in retrospective studies because of their heterogeneity.

A linear mixed model (LMM) is a parametric linear model for longitudinal data that quantifies the relationships between a continuous dependent variable and various predictor variables, providing a simple and effective way to incorporate within-subject and between-subject variations and the correlation structure of longitudinal data [21]. Analysis using a LMM is appropriate in longitudinal or repeated-measures studies, in which subjects are measured repeatedly over time or under different conditions [22]. In a LMM, fixed effects, such as sex, represent factors that are measurable and not random, while random effects represent factors that can be specified to individuals within a population and that account for the variation among individuals [23]. Therefore, the application of a mixed model is appropriate to estimate the outcomes after DHL in terms of multiple influencing factors.

Although several studies have investigated the outcomes after DHL, no study has undertaken an approach that included all or most of the important factors that could influence the results. Therefore, in the present study, we evaluated the outcomes after DHL and analyzed the factors that influence the improvement and serial change in knee motion after surgery in patients with CP, using a LMM.

## Methods

This retrospective study was approved by the institutional review board of our hospital, and informed consent was waived owing to the retrospective nature of the study.

The inclusion criteria were as follows: (1) consecutive patients with CP for less than 20 years who visited our hospital, which is a tertiary referral center for CP, between January 1995 and June 2014, (2) ambulatory patients with spastic diplegia (GMFCS level I, II and III), (3) patients who underwent distal medial hamstring lengthening plus semitendinosus transfer, (4) patients who had undergone preoperative and postoperative 3-dimensional (3D) gait analysis, and (5) patients with a minimum of 1 year of follow-up. At our hospital, postoperative gait is routinely analyzed approximately 1 to 2 years after SEMLS. From the second postoperative follow-up, we recommended regular 3D gait analysis follow-up, which was performed if the patients or their parents agreed. If a repeat surgery was performed for contracture or deformity recurrence during the follow-up period, only gait analysis data obtained before the repeat surgery were included. Exclusion criteria were as follows: (1) patients who had incomplete or missing 3D gait analysis data and (2) patients who underwent lateral hamstring lengthening or supracondylar extension osteotomy. Age at surgery, sex, duration of follow-up, GMFCS level, and details of concomitant surgeries were obtained from medical records. Intramuscular psoas lengthening (IMPL), femoral derotation osteotomy (FDO), rectus femoris transfer (RFT), tendo-Achilles lengthening (TAL), and the Strayer procedure (gastrocnemius recession) were considered relevant concomitant surgeries, which could affect sagittal plane motion during gait.

### Operative protocol

DHL, as part of a SEMLS to improve gait pattern, was performed by 2 pediatric surgeons (CYC and MSP) with 27 and 11 years of experience in pediatric orthopedics, respectively. Both surgeons followed the same treatment approach. Preoperative 3D gait analysis was used to plan the procedures. Surgical procedures were performed after considering both clinical and gait analysis findings. The indications for DHL were an increased popliteal angle and an increased knee flexion at initial contact or terminal swing. All patients underwent unilateral or bilateral DHL, which included gracilis lengthening, semitendinosus tendon transfer to the adductor magnus, and aponeurotic lengthening of the semimembranosus. In cases of concomitant RFT, the gracilis tendon was transferred to the rectus femoris tendon. In the SEMLS, FDO was performed at the intertrochanteric level of the proximal femur and TAL was performed using coronal Z-plasty. For the Strayer procedure, the gastrocnemius tendon was resected at its most distal part, and then, the resected end of the gastrocnemius tendon was sutured to the underlying soleus fascia. After the surgery, all patients were placed in a removable knee immobilizer and patients who underwent TAL or bony surgery in the foot were placed in a short leg cast. All patients had a postoperative non-weight bearing period of 3–6 weeks depending on the type of concomitant surgeries. Subsequently, the patients were referred to a local rehabilitation center to perform muscle-strengthening exercises and receive gait training.

### Acquisition of kinematic data and gait deviation index (GDI) score

3D gait analysis was performed few days before the surgery and after the surgery using a Vicon 370 system (Oxford Metrix, Oxford, United Kingdom) equipped with 7 cameras and 2 force plates. Markers were placed according to the Helen Hayes marker set [24] by 2 assistant operators under the supervision of a senior operator or by the senior operator. Patients walked barefoot on a 9-m walkway 3 times with an interval of approximately 30 s, and kinematic data were recorded. The data of the 3 trials were averaged to obtain the values of the index variables. The preoperative and postoperative kinematic variables were compared to assess the effects of DHL on knee kinematics. The GDI score [25] was calculated to determine the functional improvement in gait after surgery. A GDI score of >100 denotes a non-pathological gait, and each 10-point decrement below 100 represents 1 SD from normal kinematics. Relevant kinematic values, including knee flexion at initial contact, minimum knee flexion in the stance phase, knee range of motion (ROM), mean pelvic tilt, and GDI score, were considered outcome measures.

### Constructing an LMM

For each of 5 outcome measurements, changes in knee motion and the GDI score were adjusted for multiple factors, such as sex, age at surgery, the GMFCS level, IMPL, FDO, RFT, TAL, and the Strayer procedure as fixed effects, and follow-up duration and each subject as random effects, using an LMM. The covariance structure was considered the variance component. Restricted maximum likelihood estimation was used to produce an unbiased estimator. On examination of the individual pattern of the rate of change in the knee motion along with the follow-up duration, a LMM with a random slope and random intercept was suggested. The linearity of the follow-up duration effect was interpreted to evaluate the estimation of the 5 outcome measurements. The models were compared using the Akaike Information Criterion (AIC) and the Bayesian Information Criterion (BIC). A low AIC or BIC value is preferred in terms of model selection. All models had low AIC and BIC values, and therefore, they were considered valid for the estimation of the 5 outcome measurements.

### Statistical analysis

Descriptive statistics, such as mean and SD, were used to summarize patient demographics. The Kolmogorov–Smirnov test was used to verify the normality of the distribution of variables. The LMM was constructed to estimate the rate of change in the knee motion based on the linearity of the follow-up duration effect and with sex, age at surgery, GMFCS level, IMPL, FDO, RFT, TAL, and the Strayer procedure as covariates. The slope indicated the annual change in the estimated values obtained on 3D gait analysis. The LMM was applied to estimate the change in the values at (1) the preoperative and first postoperative evaluations and (2) subsequent postoperative evaluations according to GMFCS level (I and II vs. III).

Statistical analyses were performed using R version 3.2.5 (R Foundation for Statistical Computing, Vienna, Austria; ISBN 3–900,051–07-0; http://www.r-project.org) with the NLME package. All statistics were 2-tailed, and *p*-values <0.05 were considered significant.

## Results

**Fig. 1:**
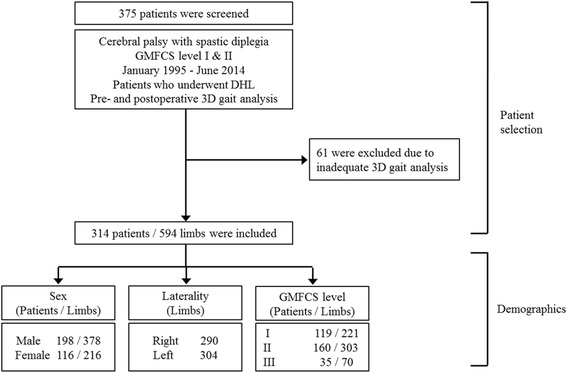
Flow chart for study inclusion

The study considered 375 patients for inclusion. However, 61 patients were excluded based on exclusion criteria. Therefore, 314 patients (594 limbs) were finally enrolled in this study. Of the patients, 198 were male and 116 were female, and 119 had GMFCS level I, 160 had GMFCS level II and 35 had GMFCS level III (Fig. 1). The mean age of the patients at the time of surgery and the mean age at the final follow-up were 7.9 ± 3.7 years (range, 3.4–20.0 years) and 10.6 ± 4.6 years (range, 5.3–26.8 years), respectively. The total number of surgical procedures performed was 1935, including DHLs (mean, 3.3 per limb), as well as 55 IMPLs, 314 FDOs, 443 RFTs, 382 TALs, and 147 Strayer procedures. Additionally, 607 preoperative and postoperative 3D gait analyses were performed. The mean follow-up duration was 2.7 ± 2.9 years (range, 1.0–14.7 years). The first postoperative 3D gait analysis was performed at a mean of 1.7 ± 1.0 years after surgery. Eighty five limbs that underwent 3D gait analysis over 3 times were included to analyze longitudinal outcomes (Table 1). The mean durations between the first and second postoperative 3D gait analyses, and between the second and third postoperative 3D gait analyses were 4.7 and 8.3 years, respectively.

**Table 1:** Patients demographics and summary of concomitant surgeries and gait parameters

	Value	
Sex (male/ female)	198 / 116	
Laterality (right/ left)	290 / 304	
Age at surgery (years)	7.9 ± 3.7 (3.4–20.0)	
Age at final follow-up (years)	10.6 ± 4.6 (5.3–26.8)	
Follow-up duration (years)	2.7 ± 2.9 (1.0–14.7)	
No. of follow-up	2 (2–4)	
Preoperative popliteal angle (degree)	58.1 ± 13.3	
Preoperative knee flexion contracture (degree)	2.2 ± 5.3	
Concomitant surgery	Limbs	
Intramuscular psoas lengthening	55 (9.3%)	
Femoral derotation osteotomy	314 (52.9%)	
Rectus femoris transfer	443 (74.6%)	
Tendo-Achilles lengthening	382 (64.3%)	
Strayer procedure	147 (24.7%)	
Gait parameters	Preoperative	Postoperative
Mean pelvic tilt (°)	16.7 ± 6.7	17.5 ± 6.1
Knee flexion at initial contact (°)	33.6 ± 11.8	24.6 ± 9.9
Minimum knee flexion in stance (°)	11.3 ± 14.1	6.5 ± 10.8
Knee range of motion (°)	47.1 ± 15.4	52.5 ± 11.9
Gait deviation index	69.9 ± 10.2	79.8 ± 9.6

**Fig. 2:**
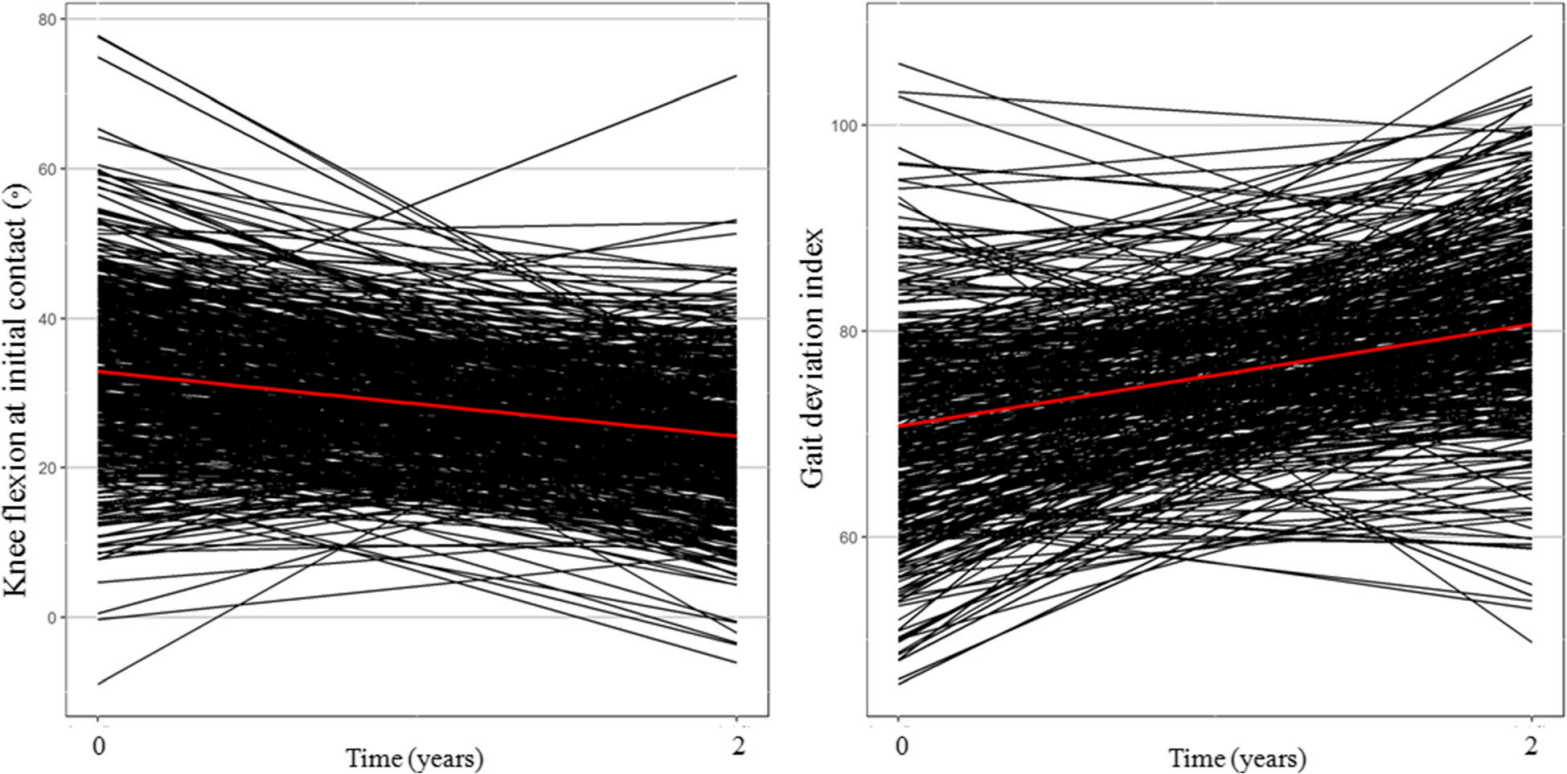
Change in knee flexion at initial contact and gait deviation index score 2 years after distal hamstring lengthening in patients with GMFCS level I and II

**Fig. 3:**
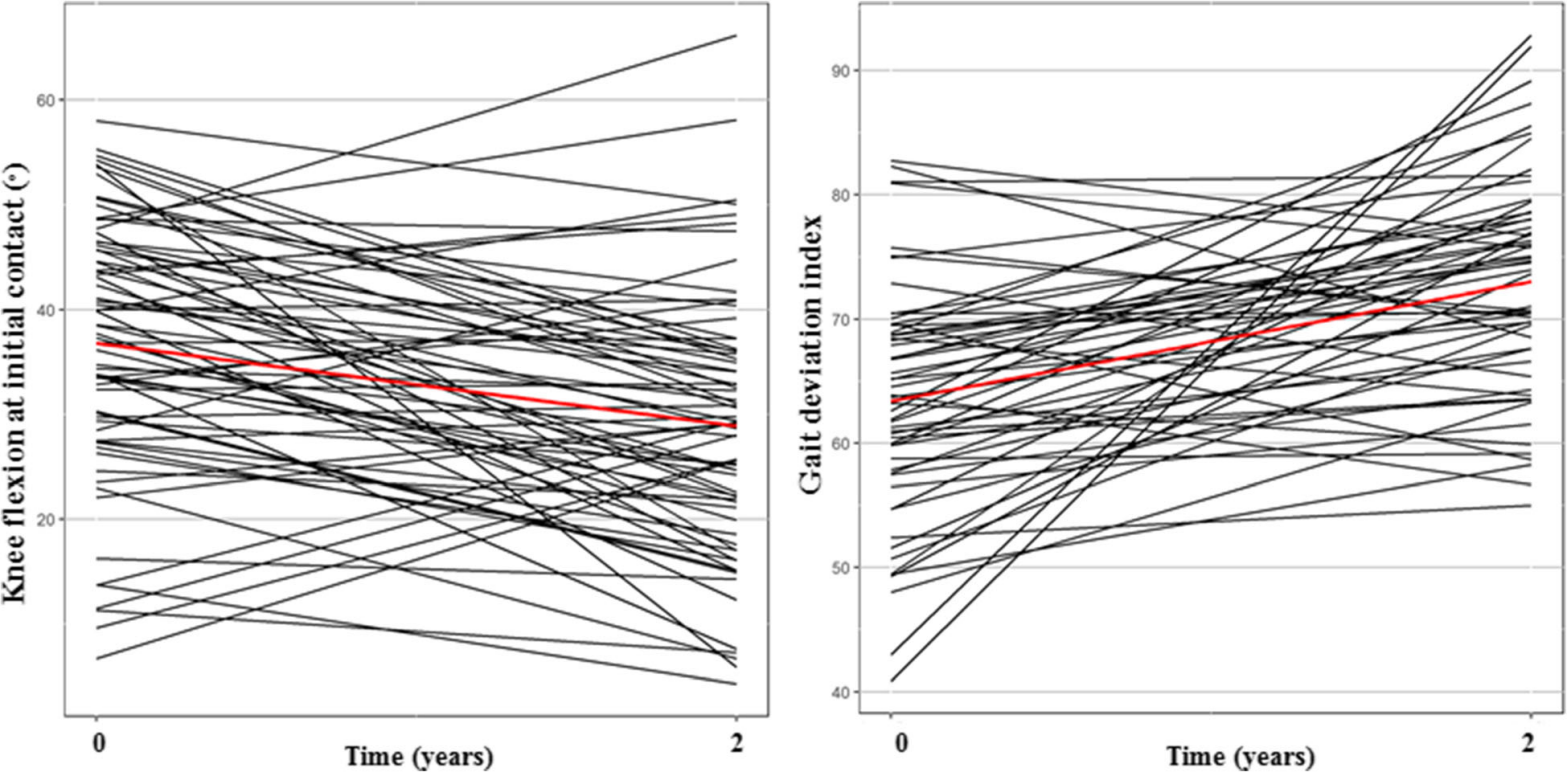
Change in knee flexion at initial contact and gait deviation index score 2 years after distal hamstring lengthening in patients with GMFCS level III

At 2 years after DHL, the estimated knee flexion at initial contact and minimum knee flexion in the stance phase were significantly decreased by 8.7° and 6.0° (both *p* < 0.001), respectively, in patients with GMFCS level I and II, and by 8.1° (*p* < 0.001) and 5.0° (*p* = 0.009), respectively, in patients with GMFCS level III. In addition, the estimated knee ROM and GDI score had significantly improved 2 year after DHL by 7.4° and 9.9 (both *p* < 0.001), respectively, in patients with GMFCS level I and II, and by 7.1° (*p* = 0.002) and 10.4 (*p* < 0.001), respectively, in patients with GMFCS level III (Figs. 2 and 3). The estimated mean pelvic tilt did not significantly changed in patients with GMFCS level I and II (*p* = 0.053) and those with GMFCS level III (*p* = 0.958) (Tables 2 and 3).

**Table 2:** Change in estimated value of sagittal kinematics and GDI score and affecting factors at 2 years after distal hamstring lengthening in patients with Gross Motor Function Classification System level I and II

	Mean pelvic tilt (°)			KF at initial contact (°)			Minimum KF in stance (°)			Knee range of motion (°)			GDI score		
	Estimation (95% CI)	SE	*p*-value	Estimation (95% CI)	SE	*p*-value	Estimation (95% CI)	SE	*p*-value	Estimation (95% CI)	SE	*p*-value	Estimation (95% CI)	SE	*p*-value
Duration	0.5 (0.0, 1.0)	0.3	0.053	−8.7 (−9.8, −7.5)	0.6	<0.001	−6.0 (−7.2, −4.8)	0.6	<0.001	7.4 (6.1, 8.7)	0.7	<0.001	9.9 (8.7, 11.2)	0.6	<0.001
Age at surgery	−0.0 (−0.2, 0.1)	0.1	0.909	−0.0 (−0.2, 0.2)	0.1	0.982	0.9 (0.6, 1.2)	0.1	<0.001	−1.2 (−1.5, −0.9)	0.1	<0.001	−0.0 (−0.3, 0.2)	0.1	0.874
Sex	1.8 (0.6, 3.0)	0.6	0.004	−0.3 (−2.1, 1.6)	1.0	0.789	−0.9 (−3.1, 1.3)	1.1	0.434	1.5 (−0.8, 3.7)	1.1	0.207	−1.2 (−3.0, 0.6)	0.9	0.203
IMPL	2.0 (0.2, 3.8)	0.9	0.030	−0.9 (−3.9, 2.2)	1.5	0.577	−0.3 (−3.8, 3.1)	1.7	0.854	−3.4 (−7.0, 0.2)	1.8	0.061	−4.3 (−7.2, −1.4)	1.5	0.004
FDO	0.5 (−0.3, 1.3)	0.4	0.200	2.4 (0.9, 4.0)	0.8	0.002	0.7 (−1.0, 2.4)	0.9	0.401	−0.2 (−2.0, 1.6)	0.9	0.831	−3.2 (−4.8, −1.7)	0.8	<0.001
RFT	0.2 (−0.8, 1.2)	0.5	0.708	1.7 (−0.1, 3.6)	1.0	0.071	−0.6 (−2.7, 1.5)	1.1	0.557	0.2 (−2.0, 2.4)	1.1	0.853	−1.9 (−4.4, 0.6)	1.3	0.141
TAL or Strayer	−0.4 (−2.2, 1.4)	0.9	0.658	2.2 (−0.7, 5.2)	1.5	0.136	1.0 (−2.4, 4.4)	1.7	0.559	−0.2 (−3.7, 3.3)	1.8	0.920	0.8 (−2.2, 3.9)	1.5	0.584

*CI* confidence interval, *SE* standard error; *KF* knee flexion, *IMPL* intramuscular psoas lengthening, *FDO* femoral derotation osteotomy, *RFT* rectus femoris transfer, *TAL* tendo-Achilles lengthening, *GDI* gait deviation index

**Table 3:** Change in estimated value of sagittal kinematics and GDI score and affecting factors at 2 years after distal hamstring lengthening in patients with Gross Motor Function Classification System level III

	Mean pelvic tilt (°)			KF at initial contact (°)			Minimum KF in stance (°)			Knee range of motion (°)			GDI score		
	Estimation (95% CI)	SE	*p*-value	Estimation (95% CI)	SE	*p*-value	Estimation (95% CI)	SE	*p*-value	Estimation (95% CI)	SE	*p*-value	Estimation (95% CI)	SE	*p*-value
Duration	0.1 (−2.0, 2.1)	1.0	0.958	−8.1 (−11.5, −4.7)	1.7	<0.001	−5.0 (−8.7, −1.2)	1.9	0.009	7.1 (2.5, 11.7)	2.3	0.002	10.4 (7.1, 13.7)	1.7	<0.001
Age at surgery	1.0 (0.3, 1.6)	0.3	0.006	0.1 (−0.8, 1.0)	0.5	0.853	0.1 (−1.1, 1.4)	0.6	0.814	−1.5 (−2.7, −0.2)	0.6	0.020	−0.5 (−1.1, 0.1)	0.3	0.125
Sex	1.9 (−2.9, 6.6)	2.4	0.438	−0.8 (−6.7, 5.0)	3.0	0.780	−6.0 (−14.5, 2.5)	4.3	0.164	2.4 (−5.5, 10.2)	4.0	0.554	−3.3 (−6.7, 0.2)	1.8	0.063
IMPL	2.9 (−2.5, 8.2)	2.7	0.294	0.9 (−6.7, 8.6)	3.9	0.810	1.3 (−8.4, 11.0)	4.9	0.794	0.7 (−9.6, 11.0)	5.3	0.894	−7.4 (−13.3, −1.5)	3.0	0.015
FDO	−0.4 (−3.7, 2.8)	1.7	0.791	4.6 (−0.2, 9.3)	2.4	0.060	5.2 (−0.7, 11.1)	3.0	0.084	−2.5 (−8.9, 3.9)	3.3	0.450	−3.2 (−6.6, 0.2)	1.8	0.068
RFT	−0.6 (−5.5, 4.3)	2.5	0.803	7.6 (0.6, 14.5)	3.6	0.033	−3.8 (−12.7, 5.2)	4.6	0.409	0.4 (−9.0, 9.8)	4.8	0.936	−5.6 (−13.0, 1.8)	3.8	0.139
TAL or Strayer	2.7 (−2.4, 7.8)	2.6	0.294	−1.0 (−7.6, 5.7)	3.4	0.779	−1.7 (−10.9, 7.4)	4.7	0.711	−2.7 (−11.7, 6.3)	4.6	0.555	1.2 (−3.4, 5.7)	2.3	0.622

*CI* confidence interval, *SE* standard error, *KF* knee flexion, *IMPL* intramuscular psoas lengthening, *FDO* femoral derotation osteotomy, *RFT* rectus femoris transfer, *TAL* tendo-Achilles lengthening; GDI, gait deviation index

In patients with GMFCS level I and II, the extent of improvement in knee flexion at initial contact was greater in patients who underwent FDO (2.4°, *p* = 0.002) than in those who did not undergo FDO. The improvement in GDI score was greater in patients who underwent IMPL (4.3, *p* = 0.004) or FDO (3.2, *p* < 0.001) than in those who did not undergo IMPL or FDO. The increase in mean pelvic tilt was less in patients who underwent IMPL (2.0°, *p* = 0.030) than in those who did not undergo IMPL (Table 2).

**Fig. 4:**
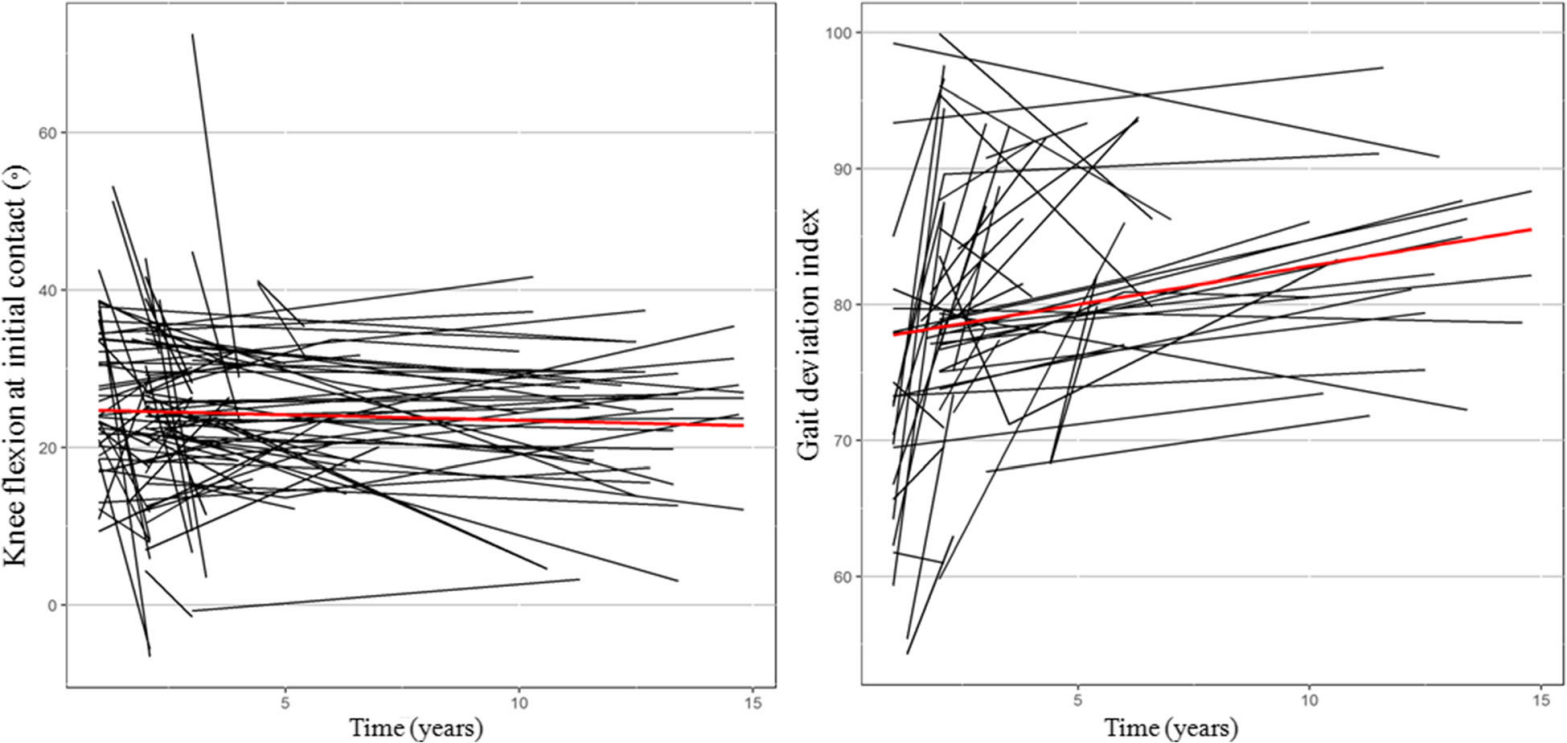
Serial change of knee flexion at initial contact and gait deviation index score throughout the follow-up duration after distal hamstring lengthening in patients with GMFCS level I and II

On evaluating the annual change of sagittal kinematics and GDI score in serial postoperative gait analyses for the patients with GMFCS level I and II, we found significant annual changes in GDI score (1.2 per year, *p* = 0.008). However, we found no significant annual change in mean pelvic tilt, knee flexion at initial contact, minimum knee flexion in the stance phase, and knee ROM (*p* = 0.543, 0.338, 0.554 and 0.755, respectively: Table 4 and Fig. 4).

**Table 4:** Serial change of sagittal kinematics and GDI score of 3-dimensiional gait analysis after distal hamstring lengthening in patients with Gross Motor Function Classification System level I and II

	Mean pelvic tilt (°)			KF at initial contact (°)			Minimum KF in stance (°)			Knee range of motion (°)			GDI score		
	Estimation (95% CI)	SE	*p*-value	Estimation (95% CI)	SE	*p*-value	Estimation (95% CI)	SE	*p*-value	Estimation (95% CI)	SE	*p*-value	Estimation (95% CI)	SE	*p*-value
Follow up (year)	−0.1 (−0.6, 0.3)	0.2	0.543	0.4 (−0.4, 1.2)	0.4	0.338	0.3 (−0.6, 1.2)	0.5	0.554	0.1 (−0.8, 1.1)	0.5	0.755	1.2 (0.3, 2.0)	0.4	0.008
Age at surgery (year)	−0.1 (−0.5, 0.4)	0.2	0.751	−0.6 (−1.4, 0.2)	0.4	0.120	0.1 (−0.8, 0.9)	0.4	0.866	−0.5 (−1.3, 0.3)	0.4	0.198	−0.7 (−1.4, 0.1)	0.4	0.098
Sex	2.4 (−0.3, 5.1)	1.4	0.081	0.8 (−3.6, 5.1)	2.2	0.735	−0.7 (−5.6, 4.2)	2.5	0.779	6.5 (1.9, 11.1)	2.4	0.006	−2.1 (−6.4, 2.1)	2.2	0.322
IMPL	1.0 (−2.1, 4.1)	1.6	0.525	0.1 (−5.2, 5.4)	0.1	0.973	0.4 (−5.6, 6.4)	3.0	0.889	−4.1 (−9.8, 1.6)	2.9	0.163	2.4 (−2.9, 7.7)	2.7	0.380
FDO	0.7 (−1.3, 2.7)	1.0	0.474	−0.8 (−5.0, 3.5)	−0.8	0.727	−1.0 (−5.6, 3.6)	2.3	0.665	2.3 (−2.3, 7.0)	2.4	0.327	−0.7 (−4.8, 3.5)	2.1	0.755
RFT	0.1 (−2.2, 2.4)	1.2	0.920	−1.8 (−6.3, 2.7)	−1.8	0.441	−2.8 (−7.8, 2.1)	2.5	0.258	5.2 (0.4, 10.1)	2.5	0.036	−0.2 (−5.2, 4.8)	2.6	0.940
TAL or Strayer	−1.3 (−4.8, 2.2)	1.8	0.462	−1.0 (−7.1, 5.2)	−1.0	0.759	1.8 (−5.1, 8.7)	3.5	0.606	−1.3 (−7.9, 5.4)	3.4	0.709	3.7 (−3.1, 10.4)	3.4	0.286

A lineal mixed model was used to estimate serial change of gait parameters after distal hamstring lengthening

*CI* confidence interval, *SE* standard error, *KF* knee flexion, *IMPL* intramuscular psoas lengthening, *FDO* femoral derotation osteotomy, *RFT* rectus femoris transfer, *TAL* tendo-Achilles lengthening, *GDI* gait deviation index

## Discussion

Although several studies have reported the outcomes after DHL, no study has considered the multiple factors that could affect the results. To our knowledge, this is the largest study to evaluate the outcomes after DHL and analyze the factors that influence the improvement and serial change in knee motion during gait in patients with CP. In the present study, sagittal knee kinematics including knee flexion at initial contact, minimum knee flexion in stance phase, and knee ROM, and GDI score significantly improved after DHL in both independently (GMFCS level I and II) and dependently (GMFCS level III) ambulatory patients. In patients with GMFCS level I and II, improvement in all sagittal knee kinematics was maintained during follow-up. Furthermore, GDI score, which represents overall gait pathology, consistently improved throughout the follow-up duration.

The present study has some limitations. First, this was a retrospective study, and therefore, the follow-up intervals varied. We used an LMM to overcome the unbalanced structure of our data set and focused on the multiple factors that could influence the improvement and annual change in knee motion after DHL. Second, the outcomes after DHL were evaluated with 3D gait analysis. Although functional improvements after DHL are important, these objective measures may not correlate well with functional and psychosocial outcomes [26]. Therefore, further studies are required on the functional and health-related quality of life outcomes. Third, no comparison group that consisted of CP patients who had not undergone surgery was included. Thus, the outcomes after DHL could have been underestimated, as the parameters in the 3D gait analysis tend to worsen in patients with CP not treated surgically [27]. Fourth, 89.0% of patients underwent triceps surae surgery (64.3% TAL and 24.7% Strayer) in this study. A TAL or Strayer operation might affect the knee kinematics. However, our analysis showed that triceps surae surgery did not affect the outcomes after DHL. We think that further study is required to investigate the effect of TAL or Strayer surgery on knee kinematics. Fifth, tibial derotation ostoetomy (TDO) or foot surgery could affect knee kinematics after DHL. However, few patients underwent TDO in this study. In addition, foot surgery was heterogeneous according to the pattern of foot deformity. Therefore, this study did not include TDO or foot surgery as concomitant surgeries for analysis. Further study is required to investigate the effect of foot surgery on knee kinematics.

DHL has been shown to reduce the stress on the knee joint and improve stance stability, which are important for normal gait [4]. However, the necessity of this procedure has been questioned because this procedure has been reported to increase anterior pelvic tilt and eventually induce crouch gait [2, 17]. In the present study, no significant change in anterior pelvic tilt 2 years after DHL was noted. In addition, no annual change in anterior pelvic tilt after surgery was noted throughout the follow-up duration. There are 2 possible reasons for this finding. First, DHL procedures in previous studies involved medial hamstring lengthening with or without lateral hamstring lengthening, while our surgical protocol involved hamstring lengthening with transfer. Semitendinosus tendon transfer eliminates its function as a knee flexor but maintains its function as a hip extensor, and the latter function can minimize increases in anterior pelvic tilt. Recent studies found better preservation of hip extension power and improved hip extension with the hamstring transfer procedure than without the procedure [10, 12]. Second, majority of the limbs (443, 74.6%) underwent RFT in the present study. A previous study reported that DHL significantly improved knee motion in patients with CP and did not increase pelvic tilt when performed with RFT [8]. Therefore, RFT might counteract the effect of DHL on pelvic tilt.

In our study, DHL, as part of a SEMLS, was effective for the treatment of flexed knee gait and the parameters of 3D gait analysis after surgery were similar to those in previous studies (Table 5). Many authors have reported short-term favorable outcomes in terms of sagittal knee joint parameters after DHL [2, 4–8, 17], and recent studies investigating the long-term outcomes of DHL showed that these improvements were maintained at over 10 years postoperatively [11, 13, 14, 18]. The present study also found that the improvement in knee flexion at initial contact, minimum knee flexion in the stance phase, and knee ROM after DHL was maintained during the follow-up duration in patients with GMFCS level I and II, although there was concern that the favorable surgical outcomes might diminished over time. Regarding an increase in knee ROM, it has been reported that a combination of RFT and DHL can improve dynamic knee function [17, 28]. In addition, the GDI score, which represents quantitative overall gait pathology, showed consistent improvement after surgery in patients with GMFCS level I and II. Therefore, we believe that DHL with lengthening and transfer can be the standard procedure in spastic diplegic CP patients with flexed knee gait and that favorable surgical outcomes can be obtained without deterioration of knee kinematics. However, only 5 patients (10 limbs) underwent 3D gait analysis more than 3 times; thus, we could not analyze the annual change in sagittal kinematics and GDI score in serial postoperative gait analyses in patients with GMFCS level III. Further study with a larger cohort is required to analyze the longitudinal outcome in patients with GMFCS level III.

**Table 5:** Previous studies investigating the outcomes after distal hamstring lengthening

Study	No. of subject	Follow-up duration (years)	Knee flexion at IC (°)			Minimum knee flexion in stance (°)			GGI or GDI^*^		
			pre	post	*p*-value	pre	post	*p*-value	pre	post	*p*-value
Dreher et al. [9]	30 (medial hamstring lengthening)	8.1 ± 1.8	36 ± 17	16 ± 12 (1Y)	<0.05	17 ± 20	0 ± 14 (1Y)	<0.05			
				22 ± 11 (2-4Y)	<0.05		9 ± 12 (2-4Y)	<0.05			
				23 ± 10 (6-12Y)	<0.05		12 ± 12 (6-12Y)	>0.05			
	9 (medial and lateral hamstring lengthening)		45 ± 15	19 ± 8 (1Y)	<0.05	35 ± 23	7 ± 12 (1Y)	<0.05			
				20 ± 12 (2-4Y)	<0.05		12 ± 16 (2-4Y)	<0.05			
				23 ± 10 (6-12Y)	<0.05		12 ± 16 (6-12Y)	<0.05			
Haumont et al. [11]	97 (185 limbs)	10.1	39.2 ± 13.4	26.6 ± 12.6	<0.01	15.6 ± 16.0	12.5 ± 14.8	>0.05	54.5 ± 15.0^*^	67.8 ± 10.4^*^	<0.01
Feng et al. [10]	20 (hamstring lengthening)	1.1 ± 0.2				31.7 ± 11.5	23.2 ± 11.6	<0.05			
	18 (hamstring transfer + lengthening)	1.1 ± 0.3				32.6 ± 11.0	18.9 ± 9.8	<0.05			
Mattos et al. [12]	18 (hamstring lengthening group)	4.3 ± 0.9				22.4 ± 13.3	9.5 ± 10.4 (1Y)	<0.05			
							10.8 ± 12.1 (final)	<0.05			
	32 (hamstring transfer + lengthening)	4.5 ± 0.9				24.9 ± 11.9	7.2 ± 10.4 (1Y)	<0.05			
							8.3 ± 12.2 (final)	<0.05			
Sung et al. [13]	29	11.8 ± 1.1	31.1 ± 12.7	26.0 ± 7.6 (1Y)	0.065	7.6 ± 13.8	2.7 ± 9.8 (1Y)	0.110	69.4 ± 11.3^*^	77.9 ± 9.5^*^ (1Y)	0.003
				23.6 ± 8.1 (10Y)	0.038		7.3 ± 10.6 (10Y)	1.000		82.2 ± 8.9^*^ (10Y)	<0.001
Ounpuu et al. [14]	22	11 ± 2	35 ± 9	23 ± 8 (1Y)	<0.001	11 ± 10	7 ± 11	>0.05			
				24 ± 9	<0.001		10 ± 11	>0.05			
Park et al. [8]	8 (16 limbs, DHL)	1.4 ± 0.8	32.9 ± 11.8	22.3 ± 8.7	0.001						
	20 (40 limbs, DHL + RFT)	1.1 ± 0.3	30.5 ± 11.7	24.9 ± 9.7	0.002						
Laracca et al. [15]	15 (right)	2.3	44.5 ± 14.2	31.7 ± 14.9	0.014	34.2 ± 19.3	24.3 ± 16.3	0.144	561 ± 194	394 ± 163	0.008
	15 (left)		41.2 ± 14.4	31.9 ± 12.0	0.054	30.8 ± 17.6	23.3 ± 15.9	0.159			
Aiona et al. [16]	28(DHL)	1.4 ± 0.5				22.8 ± 7.1	6.7 ± 8.2	<0.05			
	57 (DHL + RFT)	1.2 ± 0.5				27.1 ± 14.7	9.8 ± 11.5	<0.05			
Saraph et al. [17]	25	3.3 (3.0–3.9)	29.8	24.1	<0.001	15.2	8.2	0.006			
Chang et al. [2]	61	1.2 ± 0.4	38.3	27.4	<0.001						
Metaxiotis et al. [7]	20	3.1 (2.0–4.5)	41.5	19.1	<0.001						
Adolfsen et al. [6]	31	1.9 (0.7–6.4)	31	21	<0.001	22^a^	16^a^	<0.001			
Gough et al. [4]	13	4.2 (3.6–4.8)				31	17.7(1Y)	<0.001	1508	629 (1Y)	0.002
							22 (2Y)			794 (2Y)	
										648 (3Y)	
							20 (3Y)			652 (4Y)	
							23 (4Y)				
Ganotti et al. [18]	11	13 (11–15)	34	24 (1Y)	<0.02				335	198 (1Y)	<0.02
				26 (13Y)	<0.02					294 (13Y)	
Current study	314 (594 limbs)	2.7 ± 2.9 (1.0–14.7)	33.6 ± 11.8	24.6 ± 9.9	<0.001	11.3 ± 14.1	6.5 ± 10.8	<0.001	69.9 ± 10.2^*^	79.8 ± 9.6^*^	<0.001

*GGI* gillette gait index, A higher GGI indicates greater deviation from a normal unimpaired gait, *GDI* gait deviation index, A GDI score of 100 and above indicates nonpathological gait

^a^Present mean knee flexion in stance (°)

Several factors could affect the outcome and prognosis after DHL, such as sex, age at the time of surgery, the anatomical type of CP, and the GMFCS level. In addition, because DHL generally is performed as part of a SEMLS, the multiple concomitant procedures should be considered as influencing factors. Most studies on DHL considered bilateral limbs as independent cases. Because the correlation between right and left sides should be accounted for when analyzing data, statistical methods considering statistical independence should be used in studies involving bilateral cases. We assessed factors that influenced the outcomes after DHL via repetitive 3D gait analyses, using a LMM, and this study is the first to consider the various factors that could affect outcomes after DHL. In the present study, we found that the extent of improvement in knee flexion at initial contact was affected by whether the FDO was performed or not. However, the reason that FDO significantly affected the outcomes after DHL is unclear. Therefore, further study regarding the effects of FDO on the outcome of DHL is required. In addition, the extent of improvement in GDI score was greater in the patients who underwent concomitant surgery, including IMPAL and FDO, than in those who did not undergo these surgeries. The increase in mean pelvic tilt was also affected by whether the IMPL was performed. Therefore, we think that IMPL should be considered in patients with hip flexion contracture to prevent an increase in anterior pelvic tilt when performing DHL.

## Conclusions

Sagittal knee kinematics, including knee flexion at initial contact, minimum knee flexion in stance phase, and knee ROM, and GDI score improved after DHL in ambulatory patients with CP. In patients with GMFCS level I and II, the improvement in sagittal knee kinematics was maintained without deterioration. Furthermore, GDI score, which represents overall gait pathology, consistently improved throughout the follow-up duration. Therefore, medial hamstring lengthening with semitendinosus transfer can be the standard procedure, as part of a SEMLS, in spastic diplegic CP patients with flexed knee gait. Based on our results, physicians can predict the improvement in knee function after DHL, and inform patients and parents of the possible improvement after DHL for flexed knee gait.

## Abbreviations

CP: Cerebral palsy
DHL: Distal hamstring lengthening
FDO: Femoral derotation osteotomy
GDI: Gait deviation index
GMFCS: Gross motor function classification system
IMPL: Intramuscular psoas lengthening
LMM: Linear mixed model
RFT: Rectus femoris transfer
SEMLS: Single event multilevel surgery
TAL: Tendo-Achilles lengthening
